# LIGHT/TNFSF14 as a New Biomarker of Bone Disease in Multiple Myeloma Patients Experiencing Therapeutic Regimens

**DOI:** 10.3389/fimmu.2018.02459

**Published:** 2018-10-23

**Authors:** Giacomina Brunetti, Rita Rizzi, Giuseppina Storlino, Sara Bortolotti, Graziana Colaianni, Lorenzo Sanesi, Luciana Lippo, Maria Felicia Faienza, Anna Mestice, Paola Curci, Giorgina Specchia, Maria Grano, Silvia Colucci

**Affiliations:** ^1^Section of Human Anatomy and Histology, Department of Basic Medical Sciences, Neuroscience and Sense Organs, School of Medicine, University of Bari, Bari, Italy; ^2^Section of Hematology, Department of Emergency and Organ Transplantation, School of Medicine, University of Bari, Bari, Italy; ^3^Section of Human Anatomy and Histology, Department of Emergency and Organ Transplantation, School of Medicine, University of Bari, Bari, Italy; ^4^Paediatric Unit, Department of Biomedical Science and Human Oncology, University of Bari, Bari, Italy

**Keywords:** multiple myeloma, bone disease, LIGHT/TNFSF14, RANKL, CD14^+^/CD16^+^ monocytes

## Abstract

We have previously shown that through the production of high LIGHT levels, immune cells contribute to both osteoclastogenesis and bone destruction in Multiple Myeloma (MM)-related bone disease. With the aim of further exploring the mechanisms underlying the development of MM-related bone disease, here we focused on a possible role of LIGHT in MM patients with active bone disease despite the treatment received. We detected LIGHT over-expression by circulating CD14^+^ monocytes from MM patients still showing active bone disease, despite the treatment. In addition, we found over-expression of receptor activator of nuclear factor kappa-B ligand (RANKL), whose pro-osteoclastogenic role is well-known, in T-lymphocytes isolated from the same patients. Although the percentages of circulating osteoclast progenitors, CD14^+^CD16^+^ monocytes, were higher in all the MM patients than in the controls spontaneous osteoclastogenesis occurred only in the cultures derived from PBMCs of MM patients with unresponsive bone disease. Of note, in the same cultures osteoclastogenesis was partially or completely inhibited, in a dose-dependent manner, by the addition of RANK-Fc or anti-LIGHT neutralizing antibody, demonstrating the contribution of both LIGHT and RANKL to the enhanced osteoclast formation observed. In addition, high serum levels of TRAP5b and CTX, the two markers of osteoclast activity, were detected in MM patients with bone disease not responsive to treatment. In conclusion, our study indicates a prominent role of LIGHT in the crosstalk among osteoclasts and immune cells, co-involved together with RANKL in the pathophysiological mechanisms leading to MM-related bone disease. This TNF superfamily member may thus be a possible new therapeutic target in MM-related bone disease.

## Introduction

Multiple myeloma (MM) is a hematological malignancy that remains incurable (1). It results from the clonal expansion in the bone marrow of abnormal plasma cells, that have a reciprocal relationship with the surrounding BM microenvironment, through soluble factors and cell interactions with osteoclasts (OCs), stromal cells and osteoblasts (2). This situation leads to the development of osteolytic lesions with bone loss and, in turn, to MM expansion and progression (2, 3). MM is characterized by the production of monoclonal intact immunoglobulins or immunoglobulin free light chains, leading on to renal failure, anemia, immunosuppression, and osteolytic bone disease, that occurs in most of these patients (3). The latter is, indeed, detected in ~70% of cases at the initial diagnosis of MM; it persists even in the absence of active disease, being a major cause of morbidity and mortality in MM patients (4). Despite the improvement in treatment options, during the clinical course of the disease 80–90% of patients experience skeletal-related events (SRE) such as bone pain (70–80% of the patients), spontaneous fractures (50–60%), hypercalcemia (15%), and spinal cord compression (2–3%) (4). The development of bone lytic lesions is due to an alteration of the dynamic balance between bone-resorption and bone-formation, caused by an enhanced OC formation and activity, and an impaired osteoblast function (5, 6).

However, in the MM bone marrow microenvironment an increase in pro-osteoclastogenic factors, produced by the different cell types, such as stromal cells, lymphocytes, and other cells with an immunological role, contributes to enhance the formation of OCs, favoring the recruitment of various OC progenitors, including dendritic cells and CD14^+^CD16^+^ monocytes (7, 8). The activated OCs can also contribute to reverse the dormant state of myeloma cells (9), resulting in MM expansion and progression. In addition, the OCs play a role in maintaining an immune suppressive environment in MM (10), and the immune cells sustain the increased OC differentiation (11–14). Consistently, we found that osteoclastogenesis is supported by CD14^+^ monocytes, CD8^+^ T-cells, and neutrophils from MM-bone disease patients by means of an elevated production of the TNF superfamily member LIGHT/TNFSF14 (homologous to lymphotoxin exhibiting inducible expression and competing with Herpes Simplex Virus glycoprotein D for Herpesvirus entry Mediator [HVEM], a receptor expressed by T lymphocytes) (14). We have further demonstrated that LIGHT synergizes with receptor activator of nuclear factor kappa-B ligand (RANKL) in sustaining OC formation in MM (14). RANKL is the most closely studied pro-osteoclastogenic cytokine and its pharmacological relevance is currently recognized. Indeed, a fully human antibody to RANKL (namely Denosumab) has been developed to counteract bone resorption, OC formation, and to prevent SRE, as recently reported (15). In MM-bone disease, RANKL is the target of an innovative therapeutic approach as compared to the currently used bisphosphonates (15). Furthermore, the most important anti-myeloma agents, such as proteasome inhibitors (PIs) and immunomodulatory drugs (IMiDs), also show effects on bone cells (4, 16, 17).

Despite improvements in the depth and duration of response obtained, MM always relapses (18). In particular, patients with active bone disease together with relapsed/refractory or progressive disease (PD) pose a therapeutic challenge, prompting the need for research into new pharmacological approaches and biomarkers. Thus, with the aim of identifying possible molecules playing a role as biomarkers in bone disease of MM patients experiencing different therapeutic regimens, we investigated the expression of LIGHT and RANKL as well as their involvement in *in vitro* osteoclastogenesis.

## Patients, materials, and methods

### Patients and samples

The study included 102 patients diagnosed as having symptomatic MM experiencing 1st or 2nd line therapy. In particular, 47 patients resulted responsive at treatment response evaluation, showing a stringent complete response (sCR), a complete response (CR), a very good partial response (VGPR), or a partial response (PR); 26 patients were at first relapse and 13 at second relapse; 16 displayed disease progression (PD). The study included 50 newly diagnosed MM (NDMM) patients as control group, and 41 healthy controls matched for age and sex with the patients.

Patients and controls gave their informed consent to enter the study, performed according to the Declaration of Helsinki, and approved by the Ethics Committee of Bari University Hospital.

Patients' clinical data are reported in Table 1. All patients underwent skeletal imaging, mostly consisting of computerized tomography at diagnosis as well as at therapy response evaluations. On-going active bone disease was delineated by the occurrence of additional osteolytic lesions and/or SRE.

**Table 1:** Characteristics of patients and controls.

**Parameters**	**Newly diagnosed MM**	**Responders**	**At relapse**	**Progressive disease**	**Controls**
Number of subjects	50	47	39	16	41
Gender (M/F)	27/23	23/24	20/19	10/6	8/5
Age	62 (30–85)	64 (42–86)	69 (42–86)	68 (55–83)	67 (49–80)
Monoclonal component					
IgGk-IgGλ-IgAk-IgAλ	22-10-12-6	18-14-11-4	21-15-3-0	8-5-1-2	
ISS-stage	ISS-1: 23	ISS-1: 19	ISS-1: 16	ISS-1: 8	
	ISS-2: 7	ISS-2: 12	ISS-2: 7	ISS-2: 3	
	ISS-3: 20	ISS-3: 16	ISS-3: 16	ISS-3: 5	
Hb g/dL. median (range)	11.2 ± 0.4 (7.6–15)	12 ± 0.22 (8.8–15.4)	10.8 ± 0.37 (7.8–15.7)	11.5 ± 0.34 (8.3–16.1)	
LDH U/L. median (range)	188 ± 32.64 (129–800)	187 ± 9.49 (124–475)	181 ± 29.70 (125–807)	156 ±53.97 (122–1,503)	
Serum Creatinine mg/dL median (range)	0.92 ± 0.37 (0.60–10)	0.84 ± 0.16 (0.45–7.07)	0.93 ± 0.33 (0.47–9.42)	0.96 ± 0.08 (0.53–2.36)	
Serum Calcium mg/dL. median (range)	9.2 ± 0.09 (8.5–12)	8.9 ± 0.05 (8.6–9.6)	9.08 ± 0.09 (8.20–10)	9 ± 0.27 (9.1–11.1)	
Bone Disease	37	38	34	12	

All were inpatients at the Hematology Section, University of Bari, Medical School. MM diagnosis, and treatment response evaluation were performed according to the International Myeloma Working Group (IMWG) guidelines (19, 20). The disease was classified according to the International Staging System (ISS) (21).

As first line therapy, patients received the 1st generation proteasome-inhibitor (PI) regimens [including bortezomib, thalidomide and dexamethasone (VTD) or bortezomib, melphalan, and prednisone (VMP)]; 2nd line therapy consisted of the 2nd generation immunomodulatory drugs (IMIDs) regimens i.e., lenalidomide combined with dexamethasone (RD) and chemotherapy (CT) [bendamustine, bortezomib, and dexamethasone (BVD), doxorubicin, bortezomib, and dexamethasone (PAD)].

### Cell cultures

OCs were obtained from unfractionated peripheral blood mononuclear cells (PBMCs) of the patients with MM and the controls. PBMCs were isolated by centrifugation on a Hystopaque 1077 density gradient (Sigma Chemical, St Louis, MO), diluted to 1 × 10^6^ cells/mL in α-Minimal Essential Medium (α-MEM) and supplemented with 10% fetal bovine serum (FBS), 100 IU/ml penicillin, and 100 μg/mL streptomycin (Gibco Limited, Uxbridge, U.K.). From PBMCs, CD14^+^ and CD2^+^ cells were purified by immunomagnetic selection (Miltenyi Biotec GmbH, Bergisch Gladbach, Germany), according to the manufacturer's instruction. Additionally, to obtain fully differentiated OCs, the PBMCs were cultured for about 30 days in the presence or absence of 25 ng/mL recombinant human macrophage colony-stimulating factor (rh-MCSF) and 30 ng/ml RANKL (R&D Systems, Minneapolis, MN). For some experiments, PBMCs from MM patients were cultured in the absence or presence of anti-LIGHT mAb (0.005–500 ng/ml; R&D Systems Inc., Minneapolis, MN) or RANK-Fc (20–100 ng/ml R&D System Inc) or control anti-immunoglobulin G (IgG)-Ab. At the end of the culture period, mature OCs were identified as tartrate-resistant acid phosphatase- positive (TRAP^+^) multinucleated cells (Sigma Aldrich, Milan, Italy) containing 3 or more nuclei. Photomicrographs were obtained using a Nikon Plan Fluor 10X/0.30 dicl. The microscope was connected with a Nikon digital camera DXM 1200; the acquisition software was Lucia G version 4.61 (build 0.64) for Nikon Italy.

### Flow cytometry analysis

One hundred microliters of PB were stained with the appropriate conjugated antibody: PE-LIGHT/TNFSF14 (R&D Systems), FITC-CD4, FITC-CD8, APC-CD45, PerCP-Cy-CD14, FITC-CD14, FITC-CD16 (BD Pharmigen, San Diego, U.S.). Data were acquired using a BD Accuri™ C6 flow cytometer (Becton Dickinson Immunocytometry System, Mountain View, CA, USA) and analyzed using BD Accuri™ C6 and Kaluza software. Positivity area was determined using an isotype-matched mAb, and a total of 2,000 events for each cell sub-population were acquired.

### RNA isolation and real-time polymerase chain reaction (PCR) amplification

Freshly purified lymphomonocytes obtained from MM patients and controls were subjected to RNA extraction using spin columns (RNeasy, QIAGEN, Hilden, Germany), according to the manufacturer's instructions, to detect the expression of RANKL and LIGHT. RNA was reverse transcribed using iScript Reverse Transcription Supermix (Bio-Rad, Hercules, CA, USA). The resulting cDNA (20 μng) was subjected to quantitative PCR (qPCR) using the SsoFast EvaGreen Supermix (Bio-Rad) on an iCycler iQ5 Cromo4 (Bio-Rad). The following primer pairs were used: LIGHT S: 5′-CAGTGTTTGTGGTGGATGG-3′, AS: 5′-GGGTTGACCTCGTGAGAC-3′(NM_003807.3), GAPDH S: TCATCCCTGCCTCTACTG, GAPDH AS: TGCTTCACCACCTTCTTG (NM_002046.5).

### Western blot analysis

Proteins from CD2^+^-T cells and CD14^+^ monocytes of MM patients and controls were solubilized with lysis buffer [50 mM Tris(Tris(hydroxymethyl)aminomethane)-HCl (pH 8.0), 150 mM HCl, 5 mM ethylenediaminetetraacetic acid, 1% NP40 and 1 mM phenylmethyl sulfonyl fluoride]. The protein concentration was measured by DC™ Protein Assay (Bio-Rad, California, U.S.). Cell proteins were subjected to SDS-polyacrylamide gel electrophoresis (SDS-PAGE) and afterwards transferred to nitrocellulose membranes (Millipore, Massachusetts, U.S.). The membranes were incubated overnight at 4°C with mouse anti-LIGHT, mouse anti RANKL (Abcam, Cambridge, U.K.) and rabbit anti-total ERK (Santa Cruz Biotechnology, Texas, U.S.). After incubation with appropriate IRDye 800 cw goat anti-mouse and IRDye 800 cw goat anti-rabbit secondary Ab (LI-COR, Nebraska, U.S.), the membranes were detected on an Odissey scanner (LI-COR, Nebraska, U.S.).

### ELISA

Tartrate-resistant acid phosphatase 5b (TRAP 5b) and C-terminal telopeptide of collagen type 1 (CTX) levels in serum patients with MM and controls were detected by enzyme-linked immunosorbent assay (ELISA), according to the manufacturer's instructions (Biomedica, Vienna, Austria). The absorption was determined with an ELISA reader at 405 nm for TRAP 5b and at 450 nm for CTX-I (550 Microplate Reader, Bio-Rad); the results are expressed as mean ± SE.

### Statistical analyses

Statistical analyses were performed by ANOVA with SigmaPlot 12.0-Scientific Data Analysis and Graphing Software (SPSS, Chicago, IL). Results were considered statistically significant at *P* < 0.05.

## Results

### LIGHT expression in MM patients experiencing therapy

**Figure 1:**
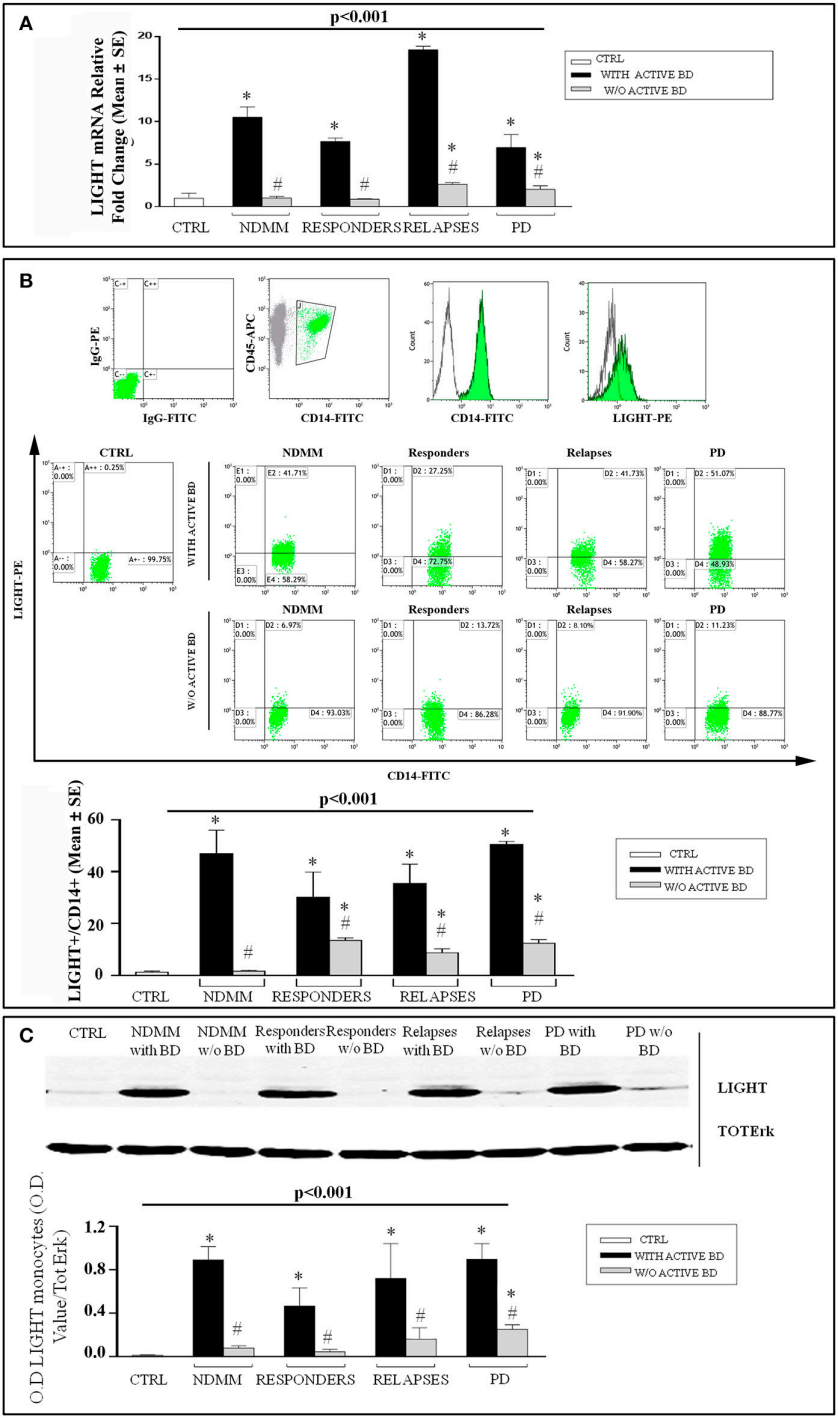
High LIGHT expression in MM patients with active bone disease experiencing therapeutic regimens. LIGHT expression was assessed by real-time PCR **(A)**, flow cytometry **(B)**, and western blotting **(C)**. In the lymphomonocytes isolated from newly diagnosed multiple myeloma (NDMM) patients with active bone disease (BD), responders, at relapse and progressive disease (PD), higher LIGHT expression was detected respect to the controls (CTRL) **(A)**. Flow cytometry graphs show IgG-FITC and IgG-PE isotype controls, CD14^+^ monocytes gated on CD45^+^ cells, single stain of a representative CD14^+^ and LIGHT^+^ cells; dot plot show LIGHT expression on CD14^+^ monocytes, from a representative CTRL and representative patients with NDMM, or responders, or at relapse or PD, without or with active BD. The histograms represent the mean values ± S.E. of all enrolled patients and CTRLs **(B)**. Western blotting analysis showing that purified monocytes from all the patients with active BD expressed high LIGHT levels while they were significantly lower in the patients without active BD and in CTRL. The optical density (O.D.) of the bands obtained by western blotting was quantified by densitometry (histograms) and normalized to Total-ERK **(C)**. The histograms represent the mean values ± S.E. of all the experiments and the blots correspond to one representative experiment. *P* < 0.001 for comparison of all groups (one-way ANOVA on ranks), and *P < 0.05 (Dunn's *post-hoc* test) vs. CTRL, ^#^*P* < 0.05 with active BD vs. w/o active BD.

Based on our previous evidence, showing that in MM some immune cells contribute to both osteoclastogenesis and bone destruction through the high levels of LIGHT, here we further explored the role of this cytokine in treated patients with MM bone disease. Thus, by means of real-time PCR, we analyzed LIGHT expression by the circulating lymphomonocytes of patients with NDMM, MM responders, at relapse, PD and of controls (Figure 1A). Interestingly, we detected the highest significant expression of LIGHT in all the MM patients shown to have active bone disease, despite treatment (*p* < 0.001, one-way ANOVA on ranks). In particular, the following mRNA fold-changes of LIGHT were found: NDMM patients (10.5 ± 1.2, *p* < 0.05), MM responder (7.6 ± 0.4, *p* < 0.05), at relapse (18.4 ± 0.5, *p* < 0.05), and PD (6.9 ± 1.5, *p* < 0.05). By contrast, lower LIGHT levels were detected in all the patients without active bone disease compared to those who have it (*p* < 0.05). Additionally in the absence of active bone disease, at relapse (2.6 ± 0.2, *p* < 0.05) and PD (2.1 ± 0.7, *p* < 0.05) patients showed a slight increase of LIGHT compared to the controls.

By flow cytometry analysis, we identified CD14^+^ monocytes as the major cellular source of LIGHT. The following percentages of LIGHT expression were found in the patients with and without bone disease: NDMM (47.9% ± 9 vs. 1.67% ± 0.3, *p* < 0.05), MM responders (30.2% ± 9.8 vs. 13.4% ± 0.95, *p* < 0.05), at relapse (35.4% ± 7.4 vs. 8.79% ± 1.5, *p* < 0.05), and PD (50.6 ± 1.1 vs. 12.4 ± 1.4, *p* < 0.05), Figure 1B. All the experiment was significant *p* < 0.001 (one-way ANOVA on ranks). These findings were supported by western blotting analysis, showing that purified monocytes from all the patients with active bone disease expressed high levels of LIGHT, whereas they were low in the patients without active bone disease (Figure 1C).

### RANKL expression in MM patients experiencing therapy

**Figure 2:**
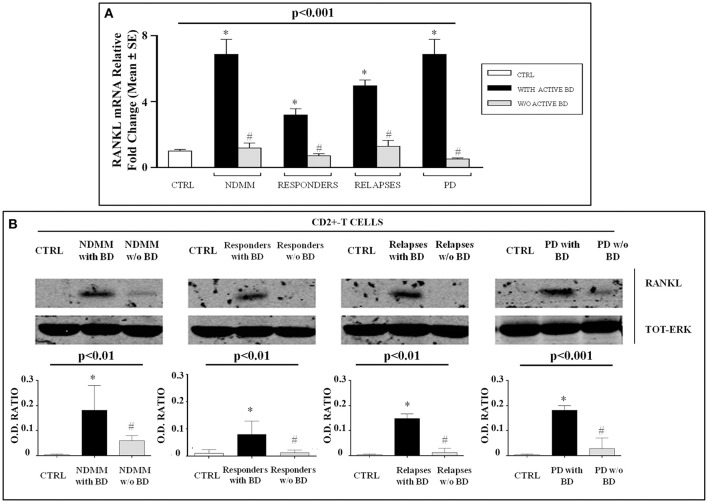
High RANKL expression in MM patients with active bone disease experiencing the therapeutic regimens. RANKL expression was assessed by real-time PCR **(A)** and western blotting **(B)**. In lymphomonocytes isolated from newly diagnosed multiple myeloma (NDMM) patients with active bone disease (BD), responders, at relapse and progressive disease (PD), higher RANKL expression was detected compared to the patients without active BD and the controls (CTRL) **(A)**. These findings were supported by western blotting analysis showing that purified CD2^+^-T cells from all the patients with active BD expressed higher RANKL levels compared to the patients without active BD and the CTRL. The optical density (O.D.) of the bands obtained by western blotting was quantified by densitometry (histograms) and normalized to Total-ERK. The histograms represent the mean values ± S.E. of all the experiments and the blot correspond to one representative experiment. *P*-value for comparison of all groups (one-way ANOVA on ranks), and *P < 0.05 (Dunn's *post-hoc* test) vs. CTRL, ^#^*P* < 0.05 with active BD vs. w/o active BD.

It is well established that RANKL is over-expressed in NDMM patients, and we have recently proved its synergic action with LIGHT in inducing OC formation. Here, we investigated the expression of RANKL in the lymphomonocytes of patients with NDMM, MM responders, at relapse, and PD. Excitingly, we found that gene levels of RANKL were significantly higher in the lymphomonocytes of all the MM patients with active bone disease experiencing therapeutic regimens compared with the patients without active bone disease and the controls, *p* < 0.001 (Figure 2A). In detail, the following RANKL mRNA fold-changes were found in the different groups of patients with active bone disease compared to the controls: NDMM (6.88 ± 0.9), MM responders (3.2 ± 0.4), at relapse (4.98 ± 0.35), and PD (6.90 ± 0.9). Since T lymphocytes are known to be a source of elevated RANKL levels, we studied the expression of this cytokine in CD2^+^-T cells isolated from the PB of all the groups of MM patients by western blotting. As expected, RANKL is over-expressed in NDMM and interestingly, it is up-regulated at protein level in all the patients with active bone disease experiencing therapeutic regimens. On the contrary, in MM patients without bone disease, RANKL expression is absent or barely detectable (Figure 2B).

### Circulating CD14^+^CD16^+^ monocytes in MM patients experiencing therapy

**Figure 3:**
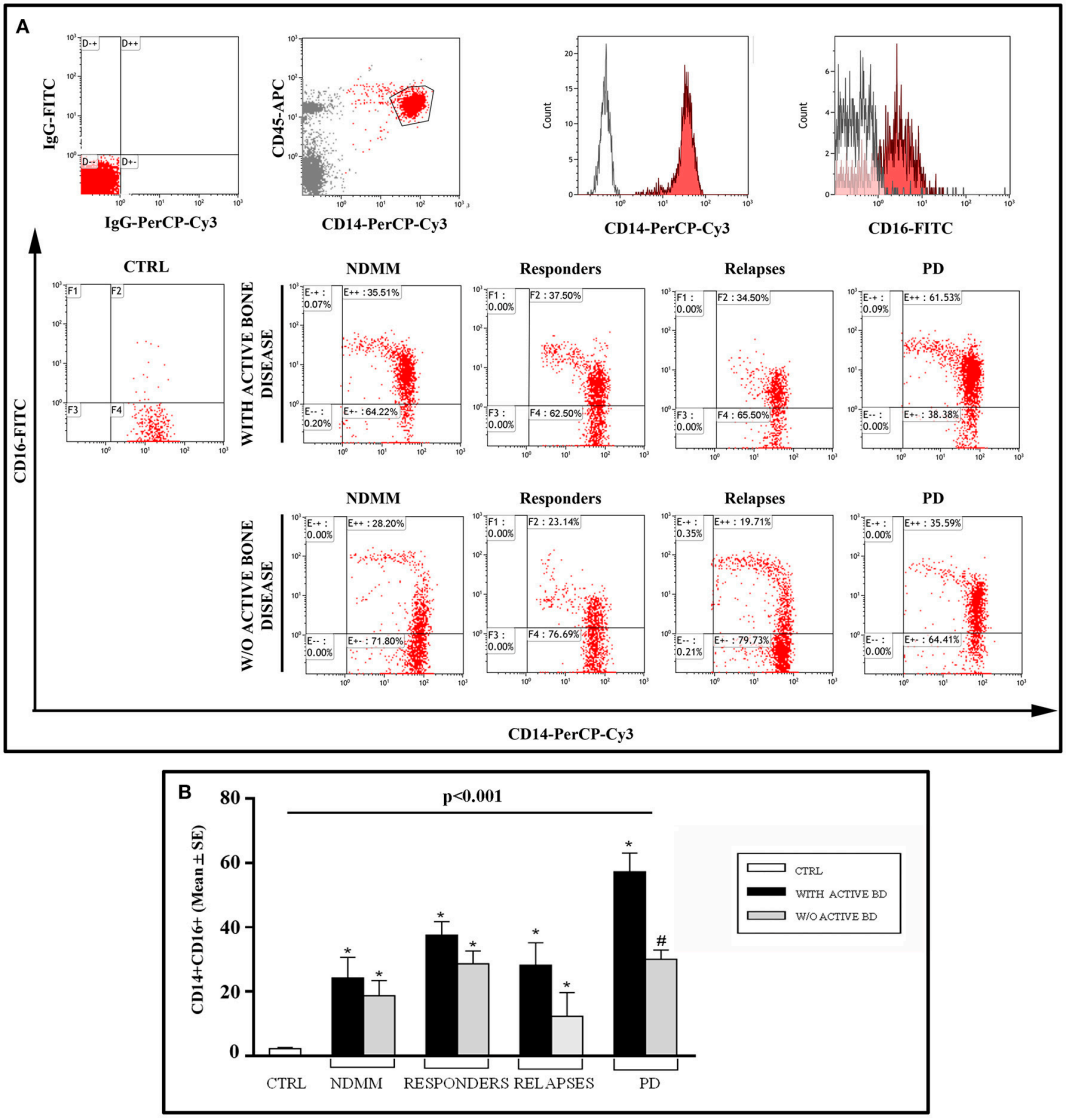
CD14^+^/CD16^+^ monocytes in MM patients and controls. **(A)** Flow cytometry graphs show IgG-FITC and IgG-PerCP-Cy3 isotype controls, CD14^+^ monocytes gated on CD45^+^ cells, single stain of a representative CD14^+^ and CD16^+^ cells; dot plots show CD14^+^/CD16^+^ monocytes, from a representative control (CTRL) and representative patients with newly diagnosed multiple myeloma (NDMM), or responders, or at relapses or progressive disease (PD) without or with active bone disease (BD). **(B)** The histograms represent the mean values ± S.E. obtained from all the enrolled controls and patients. *P* < 0.001 for comparison of all groups (one-way ANOVA on ranks), and *P < 0.05 (Dunn's *post-hoc* test) vs. CTRL, ^#^*P* < 0.05 with active BD vs. w/o active BD.

Having here shown that CD14^+^ monocytes of MM patients experiencing therapeutic regimens express high LIGHT levels, to further explore the possible alterations within the monocytes, we investigated the percentage of circulating CD14^+^CD16^+^ cells, the key monocyte subset in the commitment of OC formation. Therefore, we evaluated the percentage of these precursors in the different group of MM patients in response to treatment. A significantly high percentage of CD14^+^CD16^+^ cells was detected in all the MM patients regardless of the question of bone disease, both at diagnosis and after treatment as compared to the controls, *p* < 0.001 (Figure 3). Furthermore, the percentage of circulating CD14^+^CD16^+^ cells did not decrease in most of the patients experiencing therapeutic regimens in comparison with NDMM patients both with or without active bone disease, except for PD MM patients with active bone disease. In detail, we observed the following percentages, considering the different groups of patients with or without active bone disease: newly diagnosed MM patients (24.2% ± 6.4 vs. 18.7% ± 4.7), MM responders (37.6% ± 4.2 vs. 28.7% ± 3.9), at relapse (28.2 ± 7.0 vs. 12.3 ± 7.1), and in PD (57.3 ± 5.8 vs. 30.0 ± 2.9, *p* < 0.05).

### LIGHT and RANKL mediate *in vitro* osteoclastogenesis in MM patients experiencing therapy

**Figure 4:**
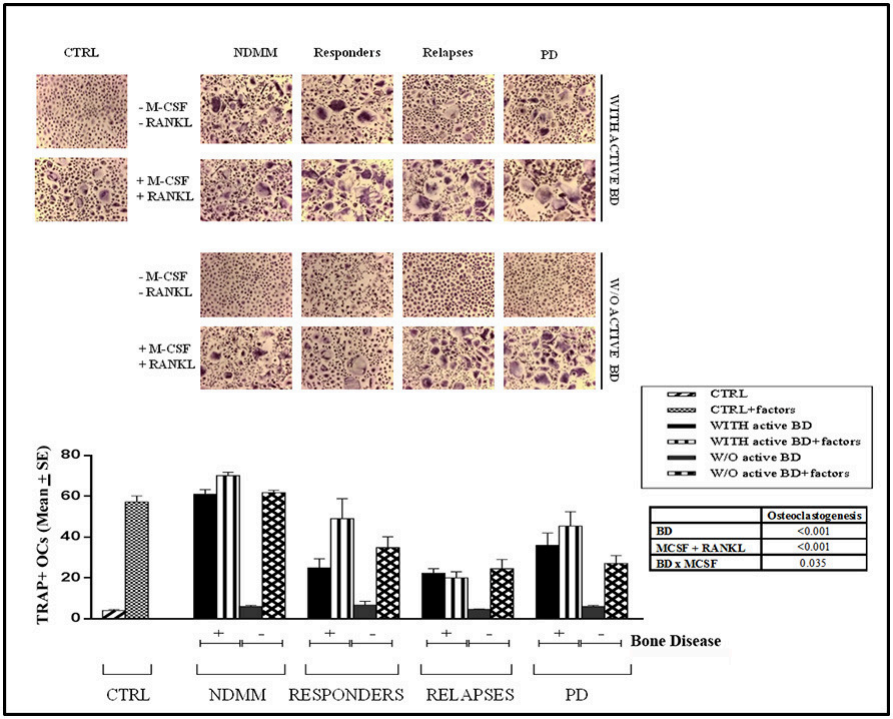
Spontaneous osteoclastogenesis in MM patients with active bone disease experiencing the therapeutic regimens. OCs were obtained from unfractionated PBMCs of controls (CTRL) and newly diagnosed multiple myeloma (NDMM) patients with or without active bone disease (BD), responders, at relapse and progressive disease (PD). Numerous, large OCs developed in the unstimulated cultures (-MCSF/RANKL) from all the MM patients with active BD, whereas rare, and small OCs were observed in the cultures from all the MM patients without active BD and CTRLs. No significant increase in OC formation was observed in PBMCs of NDMM patients with BD, at Relapse and PD due to exogenous M-CSF and RANKL (factors), whereas these cytokines significantly affected osteoclastogenesis in responder patients with active BD as well as all MM patients without BD and the CTRL. Multinucleated (>3 nuclei per cell) and TRAP^+^ cells were identified as OCs (magnification, × 200). The histograms represent the mean values ± S.E. obtained from all the enrolled controls and patients. Results reported in the table are for two-way ANOVA on treated and untreated cultures for controls and patients.

We have previously demonstrated that spontaneous osteoclastogenesis occurred in NDMM with bone disease without the addition of MCSF and RANKL, the exogenous cytokines normally necessary to induce OC formation *in vitro* (22). In this study we investigated osteoclastogenesis in MM patients experiencing therapy (Figure 4). Interestingly, we observed the spontaneous formation of a high number of large OCs from PBMCs of MM responders, at relapse and PD in patients with active bone disease (24.98 ± 4.4, 22.40 ± 2.2, and 35.97 ± 6.0, respectively). In parallel, the addition of rh-MCSF and rh-RANKL did not further increase the formation of differentiated OCs in cultures derived from patients in PD or at relapses (45.4 ± 7.0 and 20 ± 3, respectively), whereas they significantly affected osteoclastogenesis in responders (49.09 ± 9.8). Conversely, in all the patients without active bone disease osteoclastogenesis did not occur spontaneously (responders 6.7 ± 1.8, at relapse 4.75 ± 0.3 and PD 6.00 ± 0.6), and in parallel cultures the addition of rh-MCSF and rh-RANKL was absolutely necessary to induce OC formation, as it was in those from the controls (responders 34.9 ± 5.2, at relapse 24.3 ± 4.5 and PD 27.1 ± 3.8). Consistently, the results of the two-way ANOVA showed significant interaction between the bone disease and the growth factors, indicating that PBMCs reacted differently to MCSF and RANKL according the presence or absence of the bone disease.

**Figure 5:**
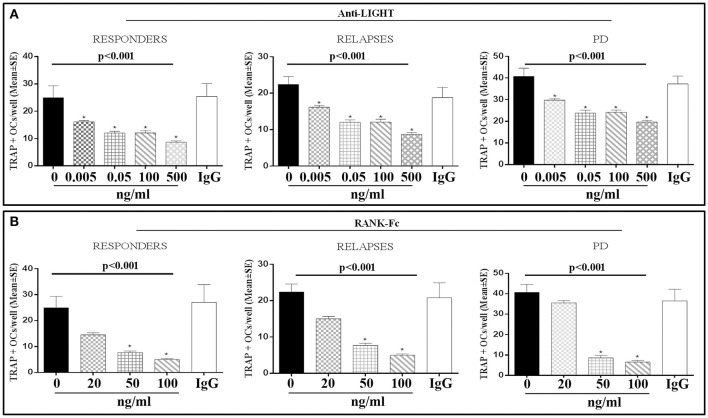
Anti-LIGHT mAb and RANK-Fc inhibit the osteoclast formation in cultures from MM active bone disease responders, relapses, and in progressive disease patients. Multinucleated and TRAP^+^ cells, differentiated from peripheral blood mononuclear cell (PBMC) cultures of responders, relapses and progressive disease (PD) patients with active bone disease (BD), were evaluated after 21 days of culture in the presence of anti-LIGHT mAb or RANK-Fc or control anti-IgG mAb. The treatment with anti-LIGHT mAb **(A)** or RANK-Fc **(B)** induced a dose-dependent inhibition of osteoclastogenesis, which was not affected by the control anti-IgG mAb. The number of multinucleated and TRAP^+^ cells, identified as OCs, are represented in the graphs as mean ± SE of all experiments performed in quintuplicate. *P* < 0.001 for comparison of all groups (one-way ANOVA on ranks), *p < 0.05 vs. CTRL (with Tukey *post-hoc* test).

Finally we demonstrated that increasing doses of anti-LIGHT (Abs) or RANK-Fc induced a dose-dependent reduction of OC numbers in PBMCs from MM patients with active bone disease in responders, at relapse and PD (Figure 5), supporting the involvement of LIGHT and RANKL in the spontaneous osteoclastogenesis. These *in vitro* findings were supported by the high *in vivo* levels of two markers of OC activity: TRAP5b and CTX (Table 2). Interestingly, we found significantly higher TRAP5b levels in all the MM patients with active bone disease than those without bone disease and controls (*p* < 0.001). In detail, we measured TRAP5b levels in patients with or without active bone disease as follows: NDMM patients (3.21 ± 0.45 U/l vs. 1.67 ± 0.26 U/l, *p* < 0.05), responders (2.30 ± 0.4 U/l vs. 1.09 ± 0.29 U/l, *p* < 0.05), at relapse (3.52 ± 0.6 U/l vs. 1.56 ± 0.09 U/l, *p* < 0.05), and PD (2.82 ± 0.38 U/l vs. 1.21 ± 0.05 U/l, *p* < 0.05). CTX serum levels resulted significantly higher in NDMM and at relapse than in the controls (*p* < 0.05). CTX levels in the patients with or without active bone disease were detected as follows: NDMM patients (0.51 ± 0.07 vs. 0.25 ± 0.02 ng/ml, *p* < 0.05), responders (0.26 ± 0.02 vs. 0.22 ± 0.05 ng/ml), at relapse (0.51 ± 0.07 vs. 0.26 ± 0.01 ng/ml, *p* < 0.05), and PD (0.23 ± 0.1 vs. 0.22 ± 0.03 ng/ml), Table 2.

**Table 2:** CTX and TRAP5b serum levels in controls and patients.

	**Controls**	**NDMM with active bone disease**	**NDMM w/o active bone disease**	**Responders with active bone disease**	**Responders w/o active bone disease**	**At relapse with active bone disease**	**At relapse w/o active bone disease**	**PD with active bone disease**	**PD w/o active bone disease**
TRAP5b	1.67 ± 0.27	3.21 ± 0.45	1.67 ± 0.26	2.30 ± 0.38	1.09 ± 0.29	3.52 ± 0.61	1.56 ± 0.09	2.82 ± 0.38	1.21 ± 0.05
*P* < 0.001	^§^*p* < 0.05	^*^p < 0.05		^*^p < 0.05		^*^p < 0.05		^*^p < 0.05	
CTX	0.32 ± 0.03	0.51 ± 0.07	0.25 ± 0.02	0.26 ± 0.02	0.22 ± 0.05	0.51 ± 0.07	0.26 ± 0.01	0.23 ± 0.1	0.22 ± 0.03
*P* < 0.006	^§^*p* < 0.05	^*^p < 0.05				^*^p < 0.05			

*P < 0.001 for comparison of all groups (one-way ANOVA on ranks), and P < 0.05 (Dunn's post-hoc test)*.

§ *Controls vs. NDMM*;

* *Patients with vs. w/o bone disease*.

Newly diagnosed multiple myeloma (NDMM); progressive disease (PD); C-terminal telopeptide of collagen type 1 (CTX); Tartrate-resistant acid phosphatase 5b (TRAP5b)

## Discussion

In this study, we demonstrated the high expression of LIGHT and RANKL in MM patients with active bone disease who experienced different therapeutic regimens. We also detected an elevated percentage of circulating CD14^+^CD16^+^ monocytes, which are the cell sub-population mainly committed to OC formation (8). Only in MM patients with active bone disease who experienced different therapeutic lines did osteoclastogenesis occur spontaneously *in vitro*. Moreover, it was inhibited by the neutralization of LIGHT and RANKL, suggesting the critical contribution of these cytokines to OC formation, and therefore to the bone disease activity. Conversely, we have low or undetectable LIGHT expression in the samples derived from MM patients without active bone disease, in which the spontaneous formation of OCs did not occur.

LIGHT is primarily produced by cells with an immunological role, and has been identified as a T-cell co-stimulatory cytokine (23–26) LIGHT constitutive expression on T-lymphocytes causes activation and expansion of these cells favoring the development of autoimmune diseases (27, 28). Moreover, our results as well as other reports sustain a key role of LIGHT in physiological and pathological bone remodeling. In particular, we found that in *LIGHT*-deficient mice (*Tnfsf14*^−/−^) an indirect effect of the immune system on bone cell activity impaired bone remodeling (29). In 2006, Edwards et al. demonstrated elevated levels of LIGHT in the sera of patients with erosive rheumatoid arthritis, and in 2014 we reported high LIGHT expression in CD14^+^ monocytes, CD8^+^ T-cells and neutrophils from NDMM-bone disease patients in both peripheral blood (PB) and bone marrow samples (14). Very recently, we demonstrated the involvement of LIGHT in pathological bone remodeling by showing high levels in sera, and on monocytes from chronic kidney disease and haemodialysis patients, in whom the impaired renal function is accompanied by an increased inflammatory state and skeletal abnormalities (30). We also found a high percentage of LIGHT-positive monocytes in Alkaptonuria, a rare metabolic disorder characterized by progressive, severe osteoarthopathy (31). In these different diseases with bone involvement, the increased LIGHT levels are primarily associated to its pro-osteoclastogenic role, as we and other authors have demonstrated (14, 30, 32). Therefore, our results showing that, despite therapy, LIGHT continues to be highly expressed, suggest a possible role for this molecule as a new biomarker of MM active bone disease.

In addition to high levels of LIGHT expression, we also detected RANKL over-expression by circulating T-cells isolated from MM patients with active bone disease even in treatment. Conversely, we found low or undetectable RANKL expression in the samples from both MM patients without active bone disease and controls. Consistently with these data, high RANKL levels were measured also in patients with Alkaptonuria, chronic kidney disease, haemodialysis, and NDMM-bone disease (12, 30, 31, 33). There are many literature reports on MM-bone disease, describing the up-regulation of RANKL in association with different mechanisms involving both cells of the osteoblastic lineage (34) and immune cells, such as T lymphocytes (12, 33, 35). Hence, in MM patients who display active bone disease concomitant with relapsed/refractory or PD despite therapy, our results demonstrating the high expression of LIGHT and RANKL are in line with the synergic effect exerted by these two cytokines on osteoclastogenesis mechanisms (14, 32).

The increased percentage of circulating CD14^+^CD16^+^ monocytes in both NDMM and in treated MM patients, regardless of the presence or absence of (active) bone disease is important for two reasons. The first is that, as recently reported, the percentage of CD14^+^CD16^+^ monocytes is quite similar in NDMM with or without bone disease, and these cells display a major pro-osteoclastogenic aptitude in cultures (8). These data suggested that in the bone marrow microenvironment the involvement of CD14^+^CD16^+^ monocytes is linked to the presence of other cells, such as the T-cells through their IL-21 over-expression (8). Therefore, we suggest that, via a similar mechanism, cells with an immunological role depending on the high production of LIGHT and RANKL can take part in the enhanced OC formation supporting bone disease in treated MM patients. Consistently, *in vitro* we found that the spontaneous osteoclastogenesis is decreased following the neutralization of LIGHT and RANKL. The second reason is based on our findings demonstrating the persistence of high CD14^+^CD16^+^ monocytes despite the therapy, thus sustaining the incurability of the disease. In fact, MM patients initially respond to the therapy but frequently relapse, and thus require additional therapy. In short, the prognosis of MM patients experiencing at least three prior lines of therapy, and becoming double refractory to IMiDs and PIs and receiving an alkylating agent, is very reduced, featuring with an event-free survival and overall survival of 5 and 13 months, respectively. To this extent, in the last stage of MM the disease becomes very complex, demonstrating a completely altered bone marrow microenvironment and profound transformation of the cells involved. For example, high levels of DKK-1, an osteoblastogenesis inhibitor, have been reported in MM patients with 3 osteolytic lesions, but its concentration decreases with the progression of bone disease (36). Similarly, among our patients, an intriguing observation was made in 2 refractory patients with active bone disease in which we measured low levels of CD14^+^CD16^+^ and LIGHT. Although this was an important observation, it is limited by the lack of statistical significance, and needs to be extended to other patients. Furthermore, it is also important to remember that in MM patients in complete remission the osteolysis usually did not repair, suggesting the persistence of the disease even when it is not detectable; this could explain the tendency to abrupt relapse. Literature data report that osteoblasts are primarily responsible for the unrepaired osteolysis (37), but it has been demonstrated that myeloma cells can be detained in a dormant state by osteoblastic cells in the endosteal niche, and this state confers dormant cells with drug resistance. Moreover, OCs can remodel the endosteal surface to liberate dormant myeloma cells from the niche and facilitate their reactivation to repopulate the tumor. Thus, myeloma cell dormancy may be a reversible state managed by the bone microenvironment, and so it is possible that a therapy targeting the cytokines and the cells of the bone marrow microenvironment could control the progress of the disease.

In conclusion, our study indicated a key role of OC and immune cell crosstalk in the pathophysiology of bone disease in MM and highlights the possibility of targeting LIGHT to preserve bone health in MM patients.

## Author's note

LS is on the Ph.D. Course of “Transplantation of Organs and Tissues and Cellular Therapies” D.E.O.T. from the Section of Human Anatomy and Histology, School of Medicine, University of Bari, Italy.

## Author contributions

GB, MG, and SC developed the concept and designed the experiments. GiuS and SB performed most experiments and analyzed data. LS performed ELISA. GB and AM performed flow cytometry. RR, PC, and GioS provided patients' samples and clinical data. GC and MF performed statistical analysis. GB, RR, MG, and SC wrote the manuscript, and all other authors commented on the manuscript.

### Conflict of interest statement

The authors declare that the research was conducted in the absence of any commercial or financial relationships that could be construed as a potential conflict of interest.
